# Eu-Substituents-Induced Modifications in the Thermoelectric Properties of the Zintl Phase Ba_1-*x*_Eu*_x_*Zn_2_Sb_2_ System

**DOI:** 10.3390/molecules30020310

**Published:** 2025-01-14

**Authors:** Daewon Shim, Junsu Lee, Aziz Ahmed, Ji Hee Pi, Myung-Ho Choi, Kang Min Ok, Kyu Hyoung Lee, Tae-Soo You

**Affiliations:** 1 Department of Chemistry, Chungbuk National University, Cheongju 28644, Chungbuk, Republic of Korea; eodnjs5375@gmail.com (D.S.); ievei4567@naver.com (J.L.); aziz.zizoou@gmail.com (A.A.); 2 Department of Materials Science and Engineering, Yonsei University, Seoul 03722, Republic of Korea; piji0627@yonsei.ac.kr (J.H.P.); khlee2018@yonsei.ac.kr (K.H.L.); 3 Department of Chemistry, Sogang University, Seoul 04107, Republic of Korea; angeliss1@naver.com (M.-H.C.); kmok@sogang.ac.kr (K.M.O.)

**Keywords:** thermoelectric material, Zintl phase, DFT calculations, BaCu_2_S_2_-type structure, electronic structure, powder X-ray diffraction, single-crystal X-ray diffraction

## Abstract

Four quaternary Zintl phase thermoelectric (TE) materials belonging to the Ba_1-*x*_Eu*_x_*Zn_2_Sb_2_ (*x* = 0.02(1), 0.04(1), 0.08(1), 0.15(1)) system were successfully synthesized using the molten Pb-flux or the conventional high-temperature reaction methods. Their crystal structures were characterized by both powder and single-crystal X-ray diffraction analyses, and all four isotypic title compounds adopted the orthorhombic BaCu_2_S_2_-type (*Pnma*, *Z* = 4, Pearson code *oP*20) structure. The radius ratio criterion, based on the cationic and anionic elements (i.e., *r*_+_/*r*_−_), was successfully verified in the title system, as in our previous reports, where *r*_+_/*r*_−_ > 1 for the BaCu_2_S_2_-type structure. A series of density functional theory calculations were performed using a hypothetical model with the idealized compositions of Ba_0.75_Eu_0.25_Zn_2_Sb_2_, and the results were compared with the ternary parental compound BaZn_2_Sb_2_ to understand the influence of Eu substituents in the Ba_1-*x*_Eu*_x_*Zn_2_Sb_2_ system. A similar overall shape of the density of states (DOS) curves and the near-constant DOS values at *E*_F_ before and after the cationic substitution suggest only marginal changes in the carrier concentration. Therefore, carrier mobility has a dominant role in rationalizing the observed variations in the electrical transport properties of the title system. Temperature-dependent TE property measurements proved that an increase in the Seebeck coefficient *S* and a decrease in electrical conductivity *σ* were observed as the Eu substituents gradually increased in the Ba_1-*x*_Eu*_x_*Zn_2_Sb_2_ system, although the overall *S* and *σ* values were lower than those in the parental compound BaZn_2_Sb_2_. The thermal conductivities of these title compounds were successfully lowered by phonon scattering, but due to the overall smaller electrical transport properties, the observed maximum *ZT* was 0.49 at 773 K for Ba_0.98(1)_Eu_0.02_Zn_2_Sb_2_.

## 1. Introduction

Thermoelectric (TE) devices can generate a significant amount of electricity from the wasted heat produced by vehicle exhausts, power plants, and other sources. To exploit this electricity for use in daily life, some issues including low energy conversion efficiencies in TE materials, thermal management problems within TE devices, TE device integration issues with heat sources should first be resolved [1,2,3,4]. The efficiency of a TE material is determined by the dimensionless figure of merit *ZT* = *S*^2^*σT*/*κ*_tot_, where *S*, *σ*, *T*, and *κ*_tot_ are the Seebeck coefficient, electrical conductivity, absolute temperature, and total thermal conductivity, respectively [3]. In particular, the *κ*_tot_ comprises two main contributions: the electronic part *κ*_elec_ related to charge carriers and the lattice part *κ*_latt_ caused by phonons. Except for *κ*_latt_, all of the TE material parameters that define *ZT* depend upon carrier concentration, *n*. Therefore, to achieve high *ZT*, the *n* of the TE material should be enhanced, and the *κ*_latt_ should be lowered [2,5].

Quite interestingly, all of the aforementioned features, necessary for the development of efficient TE materials, are presented in the Zintl phase [6,7,8]. The Zintl phase is a small bandgap semiconductor with complex crystal structures. Their *n* can properly be tuned by vacancy formation, doping, or substitution [9,10,11,12], whereas their low *κ*_tot_ is largely attributed to their complex crystal structures, which impede phonon velocities and induce different phonon scattering mechanisms [13]. Among the trigonal CaAl_2_Si_2_-type AM_2_M’_2_ (A = Ca, Sr, Ba, Eu; M = Mg, Zn, Cd; M’ = P, As, Sb) system, many compounds have been reported as TE material of interest [12,14,15,16,17,18,19,20]. On the other hand, the orthorhombic BaCu_2_S_2_-type BaM_2_M’_2_ (M = Cu, Zn; M’ = P, S, As, Se, Sb, Te) system, displaying identical atomic stoichiometry, has rarely been investigated in the TE field due to its narrow phase width and poor metallic conductivity [21,22,23]. In our previous studies on the BaCu_2_S_2_-type Ba_1-*x*_Sr*_x_*Zn_2-*y*_Cd*_y_*Sb_2_ system, we proved that the structural transformation between the BaCu_2_S_2_-type and the CaAl_2_Si_2_-type phase should be attributed to the radius ratio criterion of the cationic and anionic elements, i.e., *r*_+_/*r*_−_, with the BaCu_2_S_2_-phase preferring *r*_+_/*r*_−_ > 1 [24]. In the same study, we also demonstrated a maximum *ZT* value of 0.64 for the quaternary Ba_0.87(1)_Sr_0.13_Zn_2_Sb_2_. In another study for the BaCu_2_S_2_-type Ba_1-*x*_Sr*_x_*Zn_2-*y*_Cu*_y_*Sb_2_ system, we observed a small amount of *σ* improvement in TE power factor *PF* (*PF* = *S*^2^*σ*) upon some substitution on the parental BaZn_2_Sb_2_, which made further fine-tuning of the substituent amount necessary [25]. Lastly, we verified the aforementioned *r*_+_/*r*_−_ ratio criterion by investigating the quaternary BaZn_2-*x*_Cd*_x_*Sb_2_ system, while simultaneously obtaining the *ZT* value of 0.54 in the Zn-rich compound BaZn_1.62(2)_Cd_0.38_Sb_2_ [26].

Encouraged by the aforementioned developments of the new BaCu_2_S_2_-type Ba_1-*x*_Sr*_x_*M_2_Sb_2_ (M = Zn, Cd, Cu) systems for the TE material application, we have expanded our study to introduce the cationic Eu substituents in the parental BaZn_2_Sb_2_ system, since the Eu substitution was known to be successful for the improvement of TE properties [27]. Therefore, in this work, we conducted comprehensive experimental and theoretical investigations for the quaternary Ba_1-*x*_Eu*_x_*Zn_2_Sb_2_ system, including a series of powder and single-crystal X-ray diffraction (PXRD and SXRD) analyses for the crystal structural characterization, the density of states (DOS) curves and band structure analyses for the electronic structure analyses, and the electrical transport properties, as well as thermal conductivities measurements for the TE properties.

## 2. Results and Discussion

### 2.1. Crystal Structure Analysis

**Figure 1:**
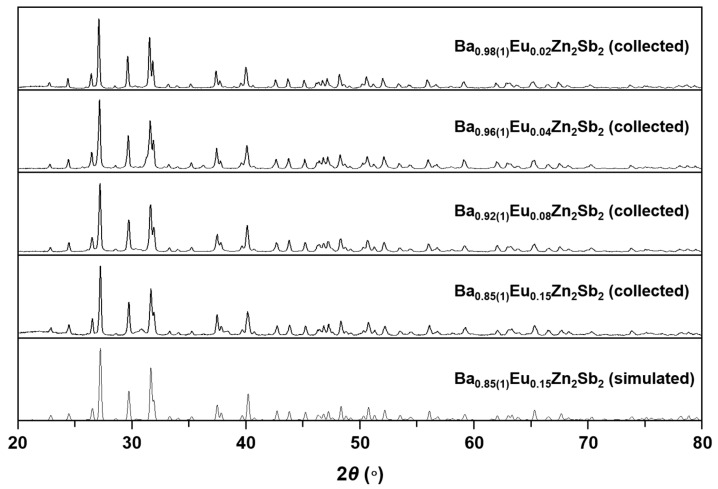
PXRD patterns of four title compounds in the Ba_1-*x*_Eu*_x_*Zn_2_Sb_2_ (0.02(1) ≤ *x* ≤ 0.15(1)) system. A simulated PXRD pattern of Ba_0.85(1)_Eu_0.15_Zn_2_Sb_2_ is also provided as a reference.

**Figure 2:**
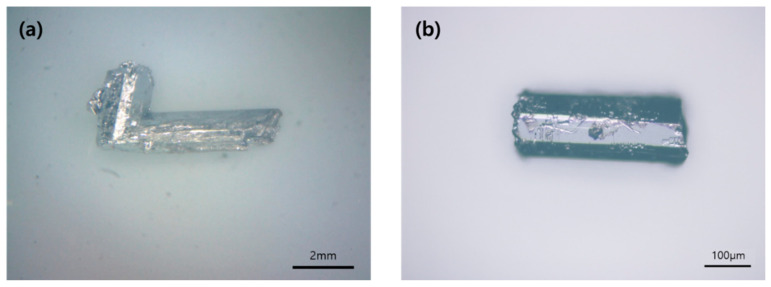
Optical Microscope images of the single crystals of (**a**) Ba_0.96(1)_Eu_0.04_Zn_2_Sb_2_ and (**b**) Ba_0.85(1)_Eu_0.15_Zn_2_Sb_2_. Scale bars are also displayed.

Four Zintl phase solid solutions in the Ba_1-*x*_Eu*_x_*Zn_2_Sb_2_ (0.02(1) ≤ *x* ≤ 0.15(1)) system have been synthesized by the molten Pb-flux method or Nb-tube method, and their crystal structures were carefully analyzed by both PXRD and SXRD analyses. The phase purities of the four title compounds were initially evaluated by indexing peaks in the collected PXRD patterns with the simulated pattern generated by the SXRD refinement result of Ba_0.85(1)_Eu_0.15_Zn_2_Sb_2_. As displayed in Figure 1, all four title compounds were proven to be single-phase products. A more comprehensive crystal structure analysis was conducted by using the SXRD data presented in Table 1, Table 2 and Table 3. The refinement results proved that all four title compounds adopted the isotypic orthorhombic BaCu_2_S_2_-type structure (*Pnma*, *Z* = 4, Pearson code *oP*20) and contained five crystallographically independent atomic sites, i.e., one Ba/Eu-mixed site, two Zn sites, and two Sb sites. Energy dispersive X-ray spectroscopy (EDS) analysis was also performed for the nicely grown bar-shaped single-crystal samples (Figure 2). Further details regarding elemental analysis and distribution mapping of the title compounds are also provided in Supplementary Figures S1–S4.

**Figure 3:**
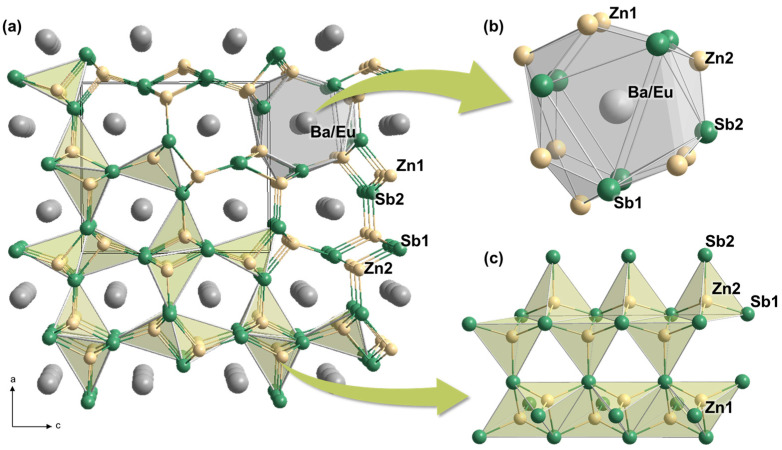
(**a**) Crystal structures of Ba_0.85(1)_Eu_0.15_Zn_2_Sb_2_ illustrated by a combination of ball-and-stick and polyhedral representations. (**b**) A Eu/Ba mixed-cationic site and (**c**) the 3D anionic frameworks of [ZnSb3/3∞3Sb1/1]  are also displayed. The tetrahedral [ZnSb_4_] building blocks are highlighted in light green polyhedra, and a unit cell is outlined with a black line. Atomic labels are also provided. Color codes: Ba/Eu mixed-site, gray; Zn, light yellow; Sb, green.

As shown in Figure 3a, the overall crystal structure of Ba_0.85(1)_Eu_0.15_Zn_2_Sb_2_ displays the three-dimensional (3D) cage-like anionic [ZnSb3/4∞3Sb1/4] frameworks, with the Ba/Eu-mixed cations filling the center of each 3D cage. In particular, these anionic frameworks consist of a one-dimensional (1D) infinite chain of [ZnSb3/3Sb1/1]∞1 by sharing two edges with their neighboring tetrahedra (Figure 3c). Moreover, each 1D chain is built by the tetrahedral [ZnSb_4_] moieties. Furthermore, the cage-shaped cationic site in the title can be described as a 16-coordinate site surrounded by nine Zn and seven Sb atoms, as displayed in Figure 3b [24].

In our recent studies of the quinary Ba_1-*x*_Sr*_x_*Zn_2-*y*_Cd*_y_*Sb_2_ and Ba_1-x_Sr_x_Zn_2-y_Cu_y_Sb_2_ systems, we rationalized, for the first time, the concept of phase selectivity between the CaAl_2_Si_2_-type and BaCu_2_S_2_-type structures in the AM_2_Sb_2_ (A = alkaline-earth metal, rare-earth metals; M = transition metal) system based on the overall radius ratio (herein referred to as *r_+_/r*_−_ratio, where *r_+_* is the average radius of cationic elements, and *r_−_* is the average radius of anionic elements) criterion [24,25]. According to our comprehensive analysis, in the case of *r_+_/r*_−_ > 1, the compounds adopted the BaCu_2_S_2_-type structure. On the other hand, if *r_+_/r*_−_ < 1, the compounds crystallized in the CaAl_2_Si_2_-type structure. In addition, in our previous studies, the overall *r_+_/r*_−_ ratio criterion depended upon both the cationic and anionic elements, with a certain amount of substituents [24,25]. However, in this work, our investigations based on the *r*_+_/*r*_−_ ratio criterion are only limited to the radius difference of the cationic elements. As relatively smaller Eu substituted for the larger Ba (*r*(Eu^2+^) = 1.20 Å, *r*(Ba^2+^) = 1.38 Å) [28] in the title Ba_1-*x*_Eu*_x_*Zn_2_Sb_2_ system, the *r_+_/r_−_* ratio gradually decreased as follows: 1.035, 1.032, 1.028, and 1.017 for Ba_0.98(1)_Eu_0.02_Zn_2_Sb_2_, Ba_0.96(1)_Eu_0.04_Zn_2_Sb_2_, Ba_0.92(1)_Eu_0.08_Zn_2_Sb_2_, and Ba_0.85(1)_Eu_0.15_Zn_2_Sb_2_, respectively. However, the *r_+_/r_−_* ratio of all four title compounds is still larger than 1, which proved that the *r_+_/r_−_* ratio criterion for the phase selectivity applies to the quaternary Ba_1-*x*_Eu*_x_*Zn_2_Sb_2_ system. Furthermore, based upon the *r_+_/r*_−_ ratio criterion, we may “reverse predict” the maximum amount of Eu substituents that maintains the BaCu_2_S_2_-type phase. Theoretically, the Ba_1-*x*_Eu*_x_*Zn_2_Sb_2_ system can maintain the BaCu_2_S_2_-type phase until ca. 28% of the Eu substituents are included, where *r_+_/r*_−_ = 1. Beyond this point, the size mismatch between the 3D cage-shaped framework and the cationic elements can escalate the repulsive interactions within the anionic frameworks in the BaCu_2_S_2_-type phase, which can eventually result in the phase transition into the more energetically favorable CaAl_2_Si_2_-type phase in the given *r_+_/r*_−_ ratio.

### 2.2. Electronic Structure Analysis

A series of DFT calculations were performed using the TB-LMTO-ASA method [29,30,31,32] to understand the effects of Ba/Eu-mixing on the electronic structure of the title compounds. It should be noted that the use of the TB-LMTO-ASA method is known to provide an underestimated bandgap when compared to those obtained by actual experiments. For practical reasons, the hypothetical structural model was designed with the idealized composition of Ba_0.75_Eu_0.25_Zn_2_Sb_2_, and the symmetry was lowered from the experimentally refined *Pnma* to its subgroup *Pm* (No. 6). The lattice parameters and atomic positions were taken from the SXRD refinement results of Ba_0.85(1)_Eu_0.15_Zn_2_Sb_2_. An additional calculation for the ternary parental compound BaZn_2_Sb_2_ was also performed for comparison purposes. Further structural details about the two hypothetical models are provided in Supplementary Table S1.

**Figure 4:**
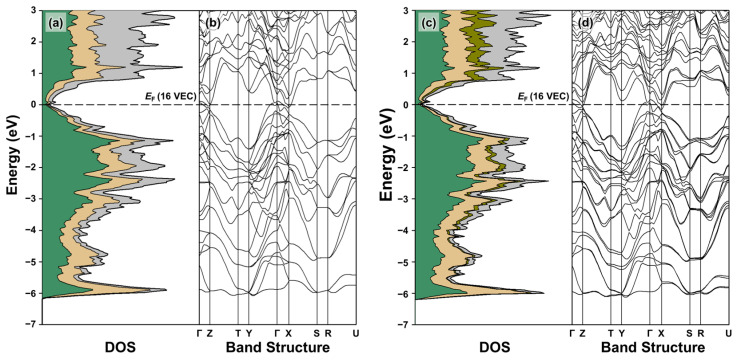
TDOS, PDOS, and band structure of BaZn_2_Sb_2_ (**a**,**b**) and Ba_0.75_Eu_0.25_Zn_2_Sb_2_ (**c**,**d**). Color codes in the DOS curves are as follows: TDOS, bold black outline; Ba PDOS, gray region; Eu PDOS, dark yellow region; Zn PDOS, light yellow region; Sb PDOS, green region. *E*_F_ (horizontal dashed line) is set as the energy reference at 0 eV, and the corresponding VEC is also displayed.

The resultant TDOS and PDOS curves and band structures are shown and compared in Figure 4. The overall shapes of the DOS curves for the two structural models closely resemble each other, including the complex orbital mixing over the entire energy window (See Figure 4a,c). In particular, below the Fermi level (*E_F_*), the strong orbital contributions from the s-states of all components are mostly observed between −11.1 and −7.0 eV (the results not shown here in order to obtain better visualization of the overall orbital distribution and resonance peak near *E_F_*). On the other hand, the orbital contributions from the p-states of the anionic Zn and Sb are dominant in the energy window between ca. −6.2 and 0 eV (E_F_), with some contributions from the cationic elements as well. In addition, a small resonance peak was observed slightly above E_F_ in the TDOS curve of the ternary BaZn_2_Sb_2_, and it became sharper and more noticeable for the idealized Eu-substituted quaternary Ba_0.75_Eu_0.25_Zn_2_Sb_3_. Furthermore, the DOS value at E_F_ showed little change upon the introduction of Eu in the parental BaZn_2_Sb_2_ system. This suggests that the carrier mobility *μ* (not the *n*) is primarily dominant for the electrical conductivity of the title Ba_1-*x*_Eu*_x_*Zn_2_Sb_2_ system. This will be further discussed in Section 2.3, where we attempt to rationalize the observed variations in this section in terms of their chemical compositions, as well as their electronic structures.

The density of states (DOS) analysis (Figure 4a,c) reveals that both BaZn_2_Sb_2_ and Ba_0.75_Eu_0.25_Zn_2_Sb_2_ possess a narrow pseudogap, indicating the absence of an actual bandgap. Previous DFT calculations for BaZn_2_Sb_2_ reported varying bandgap values (0.07, 0.2, and 0.35 eV), depending on the computational method being employed [23,33,34]. Since the TB-LMTO-ASA method tends to underestimate the bandgap size, BaZn_2_Sb_2_ likely behaves as a heavily doped semiconductor. Similarly, band structure analysis (Figure 4b,d) further reveals the lack of a clearly observable bandgap in both compounds. In the BaZn_2_Sb_2_, the valence band maxima appear near the Γ point along interval Γ-Y, while the conduction band minima are located near the X point along interval Γ-X. Moreover, the second conduction band minima exist along the interval Γ-Z. While BaZn_2_Sb_2_ and Ba_0.75_Eu_0.25_Zn_2_Sb_2_ show minimal differences in the band structure along the interval Γ-Y, the valence and conduction bands in Ba_0.75_Eu_0.25_Zn_2_Sb_2_ become closer along the Γ-Z and Γ-X intervals. Despite the narrowed energy gap, no overlap between the valence and conduction bands is observed, suggesting that Ba_0.75_Eu_0.25_Zn_2_Sb_2_ retains its heavily doped semiconducting characteristics.

### 2.3. TE Properties Measurements

To understand the influence of the Eu substitution for TE properties in the title Ba_1-*x*_Eu*_x_*Zn_2_Sb_2_ system, the temperature-dependent electrical transport properties and thermal conductivity measurements were performed for the four title compounds in the temperature range between 303 K and 797 K and between 323 K and 773 K, respectively.

**Figure 5:**
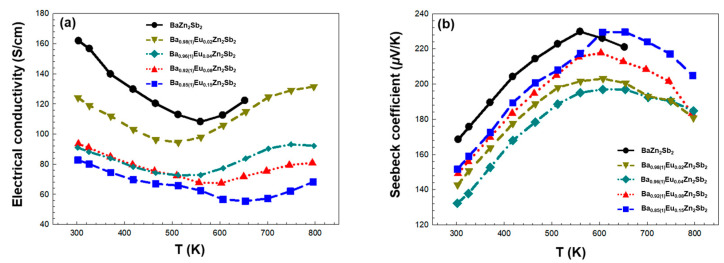
Temperature-dependent (**a**) electrical conductivity *σ* and (**b**) Seebeck coefficients *S* of the four title compounds in the Ba_1-*x*_Eu*_x_*Zn_2_Sb_2_ (0.02(1) ≤ *x* ≤ 0.15(1)) system measured between 303 and 793 K. The experimental data of the reference compound BaZn_2_Sb_2_ [24] is also plotted for comparison purposes.

First, the temperature-dependent electrical conductivities *σ* of four title compounds are illustrated in Figure 5a. All four title compounds show decreasing *σ* patterns as the temperature increases up to a “critical” temperature, which is a typical feature of the heavily doped semiconductor. Here, the increased phonon vibration obstructs the flow of free electrons, thus reducing the electrical conductivity. However, beyond this temperature, the electrical conductivities increase as the temperature increases, presenting the semiconducting characteristics, a phenomena which should be attributed to intrinsic carrier excitation across the bandgap, resulting in increased *n*. The overall temperature dependence of electrical conductivity was similar to those in previous reports on Ba_1–*x*_Sr*_x_*Zn_2–*y*_Cd*_y_*Sb_2_ and Eu_2_ZnSb_2_ Zintl phase systems [24,35]. In addition, this critical temperature gradually increased as the amount of Eu substituents increased. In other words, as the Eu concentration increased, the metallic characteristics of each title compound also increased. However, with an increasing amount of Eu, the overall *σ* values decreased, which can be attributed to the reduced mobility in our Eu-substituted samples. Basically, as more Eu is added to the Ba_1-*x*_Eu*_x_*Zn_2_Sb_2_ system, an increased structural disorder at the cationic sites results in a high carrier scattering rate. Consequently, a gradual decrease in *μ* can occur. This observation is consistent with our previous results in Section 2.2, where we predicted a major role of *μ* (not *n*) in determining the electronic transport properties of the Eu-substituted title compounds. The observed maximum *σ* values are 131, 93.5, 93.1, and 82.8 S/cm for Ba_0.98_Eu_0.02_Zn_2_Sb_2_, Ba_0.92(1)_Eu_0.08_Zn_2_Sb_2_, Ba_0.96(1)_Eu_0.04_Zn_2_Sb_2_, and Ba_0.85(1)_Eu_0.15_Zn_2_Sb_2_, respectively. Interestingly, in a previous study conducted on the Sr-substituted Ba_1-*x*_Sr*_x_*Zn_2_Sb_2_ system, we mentioned that the *σ* increase was explained in terms of the high electronegativity of Sr over Ba [24]. Due to its high electronegativity, Sr donated fewer electrons to the Zn–Sb anionic framework, resulting in an increased hole concentration. As a result, *σ* values in the investigated Ba_1-*x*_Sr*_x_*Zn_2_Sb_2_ system increased upon Sr introduction. However, in this current work, although Eu was even more electronegative than Sr (thus, more hole carriers are generated), the *σ* values decreased with the Eu substitution. This is presumably because the substantial decrease in *μ* due to the Eu-induced structural distortion was not fully compensated by the relatively smaller increase in hole concentration. This rationalization is in good agreement with the observed gradual decrease in *σ* values in our title Ba_1-*x*_Eu*_x_*Zn_2_Sb_2_ system upon the gradual introduction of Eu substituents.

Second, the temperature-dependent Seebeck coefficients *S* of the four title compounds are displayed in Figure 5b. The *S* values of all four compounds remained positive over the measured temperature range, confirming the *p*-type characteristics of all four samples. These *S* values of the four compounds increased as temperatures increased up to a certain critical temperature and then decreased. This decrease in *S* with temperature appears to be a consequence of thermally generated intrinsic carrier excitation across the bandgap and is consistent with the increase in electrical conductivity observed at the relatively higher temperature regions in Figure 5a. Except for Ba_0.96(1)_Eu_0.04_Zn_2_Sb_2_ composition, the *S* values gradually improved as the amount of Eu substituents increased. These results can be rationalized by considering that the *S* is proportional to the carrier effective mass, which in turn is related to *μ*. As the *μ* is reduced due to the Eu addition, the hole effective mass increases and eventually results in the following enhanced *S* values: $S=\frac{{8\pi }^{2}k_{B}^{2}}{{3eh}^{2}}m^{*}T{\left(\frac{\pi }{3n}\right)}^{\frac{2}{3}}$ (*k_B_* = the Boltzmann constant, *e* = the charge of electron, *h* = the Planck constant, and m^*^ = the effective mass) [36]. The maximum *S* values are 230, 218, 203, and 197 *μ*V/K for Ba_0.85(1)_Eu_0.15_Zn_2_Sb_2_, Ba_0.92(1)_Eu_0.08_Zn_2_Sb_2_, Ba_0.98_Eu_0.02_Zn_2_Sb_2_, and Ba_0.96(1)_Eu_0.04_Zn_2_Sb_2_, respectively. In particular, the *S* values for Ba_0.85(1)_Eu_0.15_Zn_2_Sb_2_ beyond 620 K are improved compared to those of the parental ternary compound BaZn_2_Sb_2_. The thermoelectric power factor *PF*, which can be calculated by *PF* = *S*^2^*σ*, was also evaluated, and the results are shown in Figure S5a. Due to the relatively smaller *S* and *σ* values of the title compounds, the overall *PF* is reduced considerably as the Eu substitution is introduced into the parental ternary phase BaZn_2_Sb_2_.

**Figure 6:**
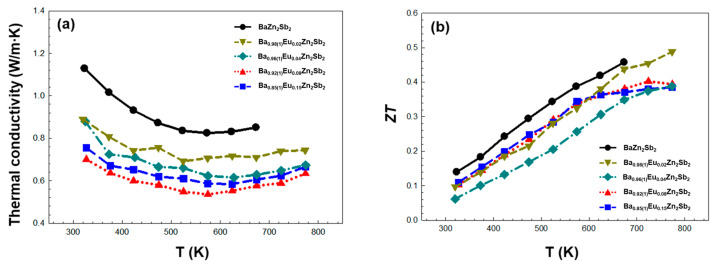
Temperature-dependent (**a**) total thermal conductivity *κ*_tot_ and (**b**) figure-of-merit *ZT* of the four title compounds in the Ba_1-*x*_Eu*_x_*Zn_2_Sb_2_ (0.02(1) ≤ *x* ≤ 0.15(1)) system measured between 323 and 773K. The experimental data of the reference compound BaZn2Sb2 [24] is also plotted for comparison purposes.

Thirdly, the temperature-dependent thermal conductivities *κ*_tot_ are provided in Figure 6a. Compared to the ternary BaZn_2_Sb_2_, the overall *κ*_tot_ of the four title compounds decreased upon the introduction of the Eu substituent in the Ba_1-*x*_Eu*_x_*Zn_2_Sb_2_ system, and this should be attributed to the enhanced cationic site disorder. This cationic site disorder caused by the Ba/Eu-mixture induces mass disorder scattering, enhances electron phonon scattering, and enables the manipulation of the phonon vibrational modes, thus causing stronger overall phonon scattering [37,38,39]. As a result, we see a significantly reduced *κ*_tot_. In particular, the observed *κ*_tot_ in our system was considerably low and even comparable to those in the previously reported thermoelectric materials with the low *κ*_tot_ [36,40]. The *κ*_tot_ values of the four title compounds show a decreasing pattern up to ca. 550 K, then beyond this point, it slightly increases. The observed minimum *κ*_tot_ values are 0.54, 0.58, 0.61, and 0.69 W/mK for Ba_0.92(1)_Eu_0.08_Zn_2_Sb_2_, Ba_0.85(1)_Eu_0.15_Zn_2_Sb_2_, Ba_0.96(1)_Eu_0.04_Zn_2_Sb_2_, and Ba_0.98_Eu_0.02_Zn_2_Sb_2_, respectively. Like the previously discussed *σ*, *κ*_tot_ also tended to decrease as the amount of Eu substituent increased. In general, the *κ*_tot_ value is expressed as the summation of the electronic term *κ*_elec_ and the lattice term *κ*_latt_. Since the *κ*_elec_ value can be calculated by the Wiedemann–Franz law (*κ*_elec_ = *LσT*, *L* = Lorenz number), the lattice term *κ*_latt_ was obtained by subtracting the *κ*_elec_ value from the *κ*_tot_ value (*κ*_latt_ = *κ*_tot_ − *κ*_elec_) [41]. By using this equation, we calculated the *κ*_elec_ values of 0.06, 0.05, 0.05, and 0.07 W/mK at 298 K for Ba_0.85(1)_Eu_0.15_Zn_2_Sb_2_, Ba_0.92(1)_Eu_0.08_Zn_2_Sb_2_, Ba_0.96(1)_Eu_0.04_Zn_2_Sb_2_, and Ba_0.98_Eu_0.02_Zn_2_Sb_2_, respectively. In addition, the *κ*_latt_ values evaluated by subtracting *κ*_elec_ from *κ*_tot_ are plotted in Supplementary Figure S5b. In this work, the contribution of *κ*_elec_ seemed to be slight, so it was confirmed that *κ*_latt_ is dominant for the overall *κ*_tot_.

Finally, the temperature-dependent figure-of-merit *ZT* values of the title compounds are provided in Figure 6b. Although the *κ*_tot_ value significantly decreased in all title compounds, the *PF* values were also low. Therefore, the positive influence of decreasing the κ_tot_ value on *ZT* was nearly compensated by *PF*. Among the four title compounds, Ba_0.98(1)_Eu_0.02_Zn_2_Sb_2_ showed the highest *ZT* value of 0.49 at 773K. In order to further improve the *ZT* of the title system, some suitable anionic substitutions can be introduced into the anionic frameworks to possibly fine-tune the hole concentration, which eventually may promote the synergetic effect [42,43,44].

## 3. Materials and Methods

### 3.1. Synthesis

The quaternary title compound Ba_0.96(1)_Eu_0.04_Zn_2_Sb_2_ was synthesized by the molten Pb-metal flux method, while three other compounds, Ba_0.98(1)_Eu_0.02_Zn_2_Sb_2_, Ba_0.92(1)_Eu_0.08_Zn_2_Sb_2_, and Ba_0.85(1)_Eu_0.15_Zn_2_Sb_2_, were synthesized by the conventional high-temperature method using a Nb ampoule (diameter = 1 cm, length = 4 cm) as a reaction container. The following reactant elements were purchased from Alfa Aesar (Ward Hill, MA, USA): Ba (rod, 99+ %), Eu (ingot, 99.9%), Zn (shot, 99.99%), Sb (shot, 99.9999%), and Pb (granules, 99.99%), and the slightly tanned surfaces of chunks of Ba and Eu elements were cleaned using a metal brush before loading. The reactants ratio used for the molten Pb-metal flux method was Ba:Eu:Zn:Sb:Pb = 0.7:0.3:2:2:10, and the mixture of reactants was loaded in an alumina crucible inside an Ar-filled glovebox (KOREAKIYON Ltd., Co., Seoul, Republic of Korea). To protect the reactants mixture from oxidation at the elevated temperature, the alumina crucible was sealed again with a fused-silica jacket under vacuum. The reactants ratio used for the conventional high-temperature reactions was Ba:Eu:Zn:Sb = (1–*x*):*x*:2:2 (*x* = 0.1, 0.3, 0.4). The reactants were cut into small pieces and loaded in the one-end-sealed Nb ampoule inside an Ar-filled glovebox, and the other end of the ampule was sealed by arc welding under the Ar atmosphere. Then, this Nb ampule was sealed again in a secondary container of a fused-silica jacket under vacuum to prevent the Nb ampoule from oxidation during the high-temperature reaction process. The reactants mixture used in the molten Pb-flux method was heated to 1223 K at the rate of 120 K/h, kept there for 24 h, and then cooled to 823 K for 92 h. At the last stage of the reaction, the reaction container was instantaneously removed from the furnace at 823 K and centrifuged for 2 min to remove the remaining molten metal flux. The reactant mixtures used for the high-temperature reaction method were heated to 1223 K at the rate of 60 K/h, kept there for 12 h, and then cooled to 893 K at the rate of 20 K/h, where the reactants were kept for 72 h. Four title compounds, Ba_0.98_Eu_0.02_Zn_2_Sb_2_, Ba_0.96(1)_Eu_0.04_Zn_2_Sb_2_, Ba_0.92(1)_Eu_0.08_Zn_2_Sb_2_, and Ba_0.85(1)_Eu_0.15_Zn_2_Sb_2_, were also re-synthesized by ball-milling followed by the hot-pressing reaction method to create the samples for the TE property measurements. A mixture of reactants, based on the targeted stoichiometry, was loaded into a stainless-steel container with two 0.5-inch and two 0.25-inch stainless steel balls inside an Ar-filled glove box. After that, the reactant mixtures were ball-milled for 2 h 30 min using the SPEX 8000M machine (SPEX SamplePrep, Metuchen, NJ, USA). During the process, the machine was stopped every 30 min to scrape and re-mix the pulverized reactant mixtures to produce homogeneous samples. After the ball-milling process, the obtained powder products were placed in a 12.5 mm graphite mold and hot-pressed under 40 MPa at 873 K for 2 h.

### 3.2. X-Ray Diffraction Analysis

The phase purities and detailed crystal structures of the four isotypic title compounds in the Ba_1-*x*_Eu*_x_*Zn_2_Sb_2_ (0.02(1) ≤ *x* ≤ 0.15(1)) system were determined by both PXRD and SXRD analyses. Four PXRD patterns were collected by the Miniflex 600 diffractometer (Rigaku Co., Tokyo, Japan) with Cu K*α*_1_ radiation (*λ* = 1.54059 Å) and plotted in Figure 1. Data collection was performed for 30 min per sample, with a step size of 0.02° in the range of 20° ≤ 2*θ* ≤ 80°. The phase purities were verified by comparing the experimentally obtained PXRD patterns to the simulated pattern generated by using the SXRD result of Ba_0.85(1)_Eu_0.15_Zn_2_Sb_2_. All four collected patterns were in good agreement with the simulated pattern, indicating the BaCu_2_S_2_-type phase.

The SXRD data of Ba_0.96(1)_Eu_0.04_Zn_2_Sb_2_ was collected at room temperature using a SMART BREEZE CCD-based diffractometer (Bruker AXS Inc., Madison, WI, USA) with Mo K*α*_1_ radiation (*λ* = 0.71073 Å). After the brief quality check for several well-grown single crystals, the best crystal was used for full data collection using Bruker’s APEX3 program (version 2019.1-0) [45]. Data reduction, integration, and unit cell parameter refinements were executed using the SAINT program (version 8.40A) [46]. The SADABS program (version 2016/2) [47] was also used to perform semi-empirical absorption corrections, based on equivalents. The SXRD data of Ba_0.98(1)_Eu_0.02_Zn_2_Sb_2_, Ba_0.92(1)_Eu_0.08_Zn_2_Sb_2_, and Ba_0.85(1)_Eu_0.15_Zn_2_Sb_2_ were collected using synchrotron radiation (*λ* = 0.65000 Å) on a MX225HS detector (Rayonix, Evanston, IL, USA) at BL2D SMC in the Pohang Accelerator Laboratory. Data collection was conducted using the PAL BL2D-SMDC program [48], and HKL3000sm (version 715) [49] was applied for cell refinement, reduction, and absorption correction. All SXRD data sets indicated the orthorhombic *Pnma* space group (No. 62). The detailed crystal structures, including the atomic positions accompanied by anisotropic displacement parameters (ADPs), the extinction coefficients, and the mixed ratio of Ba and Eu, were refined to convergences using full-matrix least-squares methods on *F*^2^. For the standardization of atomic positions, the STRUCTURE TIDY program (https://www.platonsoft.nl; accessed on 14 December 2024) [50] was used during the last step of structure refinement. More detailed crystallographic data, including atomic positions with ADP values and several selected bond distances, are presented in Table 1, Table 2 and Table 3. All these crystallographic data can be obtained free of charge from the Cambridge Crystallographic Data Center via www.ccdc.cam.ac.uk/data_request/cif (accessed on 10 January 2025). The depository numbers are as follows: CCDC-2405340 for Ba_0.98(1)_Eu_0.02_Zn_2_Sb_2_, CCDC-2405341 for Ba_0.96(1)_Eu_0.04_Zn_2_Sb_2_, CCDC-2405342 for Ba_0.92(1)_Eu_0.08_Zn_2_Sb_2_, and CCDC-2405343 for Ba_0.85(1)_Eu_0.15_Zn_2_Sb_2_.

### 3.3. Electronic Structure Calculations

To understand the overall electronic structure of the title compounds, a series of DFT calculations were performed using the TB-LMTO47 method with the atomic sphere approximation (ASA) [29,30,31,32]. Two hypothetical structural models with the idealized compositions of BaZn_2_Sb_2_ and Ba_0.75_Eu_0.25_Zn_2_Sb_2_ were designed, and the DOS curves and band structures were thoroughly analyzed. The symmetry of these models was lowered from the experimentally obtained space group *Pnma* (No. 62) to its subgroup *Pm* (No. 6) to accommodate the idealized chemical compositions in each model. Further detailed crystallographic information, including the lattice parameters and the atomic positions, was extracted from the SXRD refinement results of BaZn_2_Sb_2_ and Ba_0.85(1)_Eu_0.15_Zn_2_Sb_2_, respectively. The structural details of these two models are provided in Supplementary Table S1. All the space in a unit cell is filled with overlapping Wigner−Seitz (WS) atomic spheres, and all relativistic effects (except spin−orbit coupling) were taken into account using a scalar relativistic approximation [51]. Each WS sphere is regarded to contain the symmetrically spherical potential, and the combined correction was applied for the overlapping regions. The radii of each WS sphere were calculated automatically to ensure that the overlapping potential exhibited the best approximation to the full potential [51]. The WS radii used for the two models are as follows: Ba, 2.462 Å; Zn, 1.489−1.572 Å; Sb, 1.734−1.787 Å for BaZn_2_Sb_2_; and Ba, 2.444−2.445 Å; Eu, 2.444 Å; Zn, 1.486−1.570 Å; Sb, 1.732−1.791 Å for Ba_0.75_Eu_0.25_Zn_2_Sb_2_. The basis sets included 6s, 6p, 5d, and 4f orbitals for Ba; 6s, 6p, and 5d orbitals for Eu; 4s, 4p, and 3d orbitals for Zn; and 5s, 5p, 5d, and 4f orbitals for Sb. The Löwdin downfolding technique was applied for the Ba 6p, Eu 6p, Sb 5d, and 4f orbitals [52]. The self-consistent charge density was obtained using 360 irreducible *k*-points in the Brillouin zone for both structure models [53].

### 3.4. Thermogravimetric (TGA) Analysis

The thermal stabilities of the four title compounds, Ba_0.98_Eu_0.02_Zn_2_Sb_2,_ Ba_0.96(1)_Eu_0.04_Zn_2_Sb_2,_ Ba_0.92(1)_Eu_0.08_Zn_2_Sb_2_, and Ba_0.85(1)_Eu_0.15_Zn_2_Sb_2_, were checked by TGA using a TG 209 F1 Libra device (Netzsch-Gerätebau GmbH, Selb, Germany). A total of 20 mg of the pulverized sample was placed on an alumina pan and heated to 1173 K at a rate of 10 K/min under the continuous N_2_ flow condition. After that, the sample was naturally cooled down to room temperature. The TGA analysis proved that all four title compounds were thermally stable up to ca. 800 K (see Supplementary Figure S6).

### 3.5. EDS Analysis

Elemental analysis, including distribution mapping for the four title compounds, was performed by EDS using an ULTRA Plus field-emission (Carl Zeiss, Oberkochen, Germany) scanning electron microscope (SEM) system with an acceleration voltage of 30 kV. Several well-grown bar-shaped single crystals obtained using the molten Pb-flux method (See Figure 2) were carefully selected and placed on an aluminum puck with double-sided conductive carbon tapes under the Ar atmosphere. EDS analysis indicated comparable compositional results for Ba_0.95_Eu_0.01_Zn_2.06_Sb_1.97_, Ba_0.98_Eu_0.06_Zn_1.97_Sb_2.00_, Ba_0.91_Eu_0.10_Zn_2.00_Sb_1.99_, and Ba_0.83_Eu_0.21_Zn_1.95_Sb_2.02_ to the SXRD refinement results for Ba_0.98(1)_Eu_0.02_Zn_2_Sb_2_, Ba_0.96(1)_Eu_0.04_Zn_2_Sb_2_, Ba_0.92(1)_Eu_0.08_Zn_2_Sb_2_, and Ba_0.85(1)_Eu_0.15_Zn_2_Sb_2_, respectively. Detailed EDS analysis and distribution mapping results are provided in Supplementary Figures S1–S4.

### 3.6. Electrical Transport Property Measurement

Four disk-shaped title compounds, which were synthesized by ball-milling, followed by hot-pressing, were prepared into a bar shape (3 mm × 3 mm × 10 mm) for the electrical transport property measurements. The densities of these samples were proven to be higher than 90%, according to the geometric density measurement method. The longer direction of each bar-shaped sample coincided with the direction in which the properties were measured. The electrical conductivity *σ* and the Seebeck coefficient *S* were simultaneously measured between 303 and 793 K under the He atmosphere using a ZEM-3 instrument system (ULVAC-RIKO Inc., Yokohama, Japan).

### 3.7. Thermal Conductivity Measurement

Thermal diffusivity was measured for the four disk-shaped title compounds under an inert gas atmosphere from 323 to 773 K using a LFA 457 HyperFlash instrument (Netzsch-Gerätebau GmbH, Selb, Germany). The measurement was performed using a flash diffusion method in which the front surface of the disk was irradiated with a short laser burst, and the temperature change on the rear surface was recorded and analyzed using an IR detector. The thermal conductivity *κ*_tot_ was evaluated using the equation *κ*_tot_ = *DC*_p_*ρ* (*D* = thermal diffusivity, *C*_p_ = heat capacity, and *ρ* = density) [54]. In this work, the Dulong−Petit value (3*R*/atom, *R* = gas constant) was exploited for *C*_p_. The *κ*_tot_ was regarded as the sum of the lattice *κ*_latt_ and the electronic *κ*_elec_ thermal conductivities [54]. *κ*_elec_ was calculated using the Wiedemann—Franz law (*κ*_elec_ = *LσT*, *L* = the temperature-dependent Lorenz number), and the *L* value was estimated using the single-parabolic band model from the temperature-dependent *S* [36]. Therefore, *κ*_latt_ was obtained from the simple equation *κ*_latt_ = *κ*_tot_−*κ*_elec_ and is plotted in Supplementary Figure S5b.

## 4. Conclusions

Four new Zintl phase solid solutions in the Ba_1-*x*_Eu*_x_*Zn_2_Sb_2_ (0.02(1) ≤ *x* ≤ 0.15(1)) system were obtained using the molten Pb metal-flux or the conventional high-temperature reaction methods. Their crystal structures were characterized by PXRD and SXRD, and all four title compounds adopted the BaCu_2_S_2_-type phase with the orthorhombic *Pnma* space group. The overall crystal structure of the isotypic title compounds can be described as an assembly of (1) the 3D anionic [ZnSb3/4∞3Sb1/4] frameworks formed by the interconnection of four neighboring 1D [ZnSb3/3∞1Sb1/1] chains and (2) the mixed cationic elements filling the cage-shaped voids within these frameworks. In addition, the applicability of the previously outlined radius ratio (*r*_+_/*r*_−_) criterion, which determines the structure-type selectivity between the CaAl_2_Si_2_-type and BaCu_2_S_2_-type phases, was strongly confirmed in the title system. A series of DFT calculations using hypothetical structure models revealed that the Ba_1-*x*_Eu*_x_*Zn_2_Sb_2_ system possesses heavily doped semiconducting characteristics, and the variations in transport properties upon Eu substitution should be explained in terms of changes in carrier mobilities. The temperature-dependent TE property measurements proved that despite the significantly lowered *κ*_tot_, due to the relatively smaller *S* and *σ* values, the *ZT* values of the four Eu-substituted title compounds either decreased or slightly increased, with the maximum value of 0.49 at 773 K. We expect that the suitable anionic substitution may independently optimize the *n*, leading to overall enhanced TE properties.

## Figures and Tables

**Table 1:** SXRD data and structure refinement results for the Ba_1-*x*_Eu*_x_*Zn_2_Sb_2_ (0.02(1) ≤ *x* ≤ 0.15(1)) system.

Empirical Formula	Ba_0.98(1)_Eu_0.02_Zn_2_Sb_2_	Ba_0.96(1)_Eu_0.04_Zn_2_Sb_2_	Ba_0.92(1)_Eu_0.08_Zn_2_Sb_2_	Ba_0.85(1)_Eu_0.15_Zn_2_Sb_2_
Crystal system	orthorhombic			
Space group	*Pnma* (No. 62)			
Unit cell dimensions (Å)	*a =* 10.547(2)	*a* = 10.5449 (3)	*a =* 10.535(2)	*a =* 10.524(2)
	*b* = 4.4990(9)	*b* = 4.4973 (2)	*b* = 4.4920(9)	*b* = 4.4820(9)
	*c* = 11.640(2)	*c* = 11.6391 (4)	*c* = 11.632(2)	*c* = 11.626(2)
Volume (Å^3^)	552.33(19)	551.97(3)	550.46(19)	548.38(19)
*d*_calcd_ (g/cm^3^)	6.155	6.163	6.187	6.223
Data/restraints/parameters	904/0/32	1163/0/33	973/0/32	977/0/32
*R* indices *^a^* (*I* > 2*σ*(*I*))	*R*_1_ = 0.0245	*R*_1_ = 0.0238	*R*_1_ = 0.0231	*R*_1_ = 0.0213
	*wR*_2_ = 0.0627	*wR*_2_ = 0.0418	*wR*_2_ = 0.0612	*wR*_2_ = 0.0507
*R* indices *^a^* (all data)	*R*_1_ = 0.0274	*R*_1_ = 0.0331	*R*_1_ = 0.0246	*R*_1_ = 0.0233
	*wR*_2_ = 0.0639	*wR*_2_ = 0.0432	*wR*_2_ = 0.0619	*wR*_2_ = 0.0515
Goodness of fit on *F*^2^	0.990	1.283	1.031	1.028
Largest diff. peak/hole (e/Å^3^)	1.943/−1.628	1.243/−1.630	1.913/−1.427	1.178/−1.458

*^a^ R*_1_ = Σ**||***F*_o_**|**−**|***F*_c_**||**/Σ**|***F*_o_**|**; *wR*_2_ = {Σ[*w*(*F*_o_^2^−*F*_c_^2^)/Σ[*w*(*F*_o_^2^)^2^]]^1/2^, where *w* = 1/[*σ*^2^*F*_o_^2^+(A−*P*)^2^+B−*P*], in which *P* = (*F*_o_^2^ +2*F*_c_^2^)/3, and *A* and *B* are weight coefficients.

**Table 2:** Atomic coordinates and equivalent isotropic displacement parameters (*U*_eq_ *^a^*) from the SXRD refinements for the Ba_1-*x*_Eu*_x_*Zn_2_Sb_2_ (0.02(1) ≤ *x* ≤ 0.15(1)) system.

Atom	Wyckoff Site	Occupation	*x*	*y*	*z*	*U*_eq_ *^a^* (Å^2^)
Ba_0.98(1)_Eu_0.02_Zn_2_Sb_2_						
Ba/Eu	4*c*	0.98(1)/0.02	0.2457(1)	^1^/_4_	0.3206(1)	0.0082(2)
Zn1	4*c*	1	0.0541(1)	^1^/_4_	0.6170(1)	0.0096(2)
Zn2	4*c*	1	0.0938(1)	^1^/_4_	0.0479(1)	0.0093(2)
Sb1	4*c*	1	0.3466(1)	^1^/_4_	0.0352(1)	0.0047(2)
Sb2	4*c*	1	0.4763(1)	^1^/_4_	0.6644(1)	0.0056(2)
Ba_0.96(1)_Eu_0.04_Zn_2_Sb_2_						
Ba/Eu	4*c*	0.96(1)/0.04	0.2458(1)	^1^/_4_	0.3203(1)	0.0151(2)
Zn1	4*c*	1	0.0545(1)	^1^/_4_	0.6169(1)	0.0160(2)
Zn2	4*c*	1	0.0936(2)	^1^/_4_	0.0480(1)	0.0156(2)
Sb1	4*c*	1	0.3463(1)	^1^/_4_	0.0350(1)	0.0111(1)
Sb2	4*c*	1	0.4762(1)	^1^/_4_	0.6647(1)	0.0119(1)
Ba_0.92(1)_Eu_0.08_Zn_2_Sb_2_						
Ba/Eu	4*c*	0.92(2)/0.08	0.2461(1)	^1^/_4_	0.3197(1)	0.0065(2)
Zn1	4*c*	1	0.0549(1)	^1^/_4_	0.6165(2)	0.0072(2)
Zn2	4*c*	1	0.0930(1)	^1^/_4_	0.0478(1)	0.0069(2)
Sb1	4*c*	1	0.3457(1)	^1^/_4_	0.0344(1)	0.0028(2)
Sb2	4*c*	1	0.4761(1)	^1^/_4_	0.6651(1)	0.0035(2)
Ba_0.85(1)_Eu_0.15_Zn_2_Sb_2_						
Ba/Eu	4*c*	0.85(1)/0.15	0.2467(1)	^1^/_4_	0.3185(1)	0.0071(1)
Zn1	4*c*	1	0.0560(1)	^1^/_4_	0.6157(1)	0.0079(2)
Zn2	4*c*	1	0.0917(1)	^1^/_4_	0.0474(1)	0.0073(2)
Sb1	4*c*	1	0.3445(1)	^1^/_4_	0.0332(1)	0.0034(1)
Sb2	4*c*	1	0.4757(1)	^1^/_4_	0.6661(1)	0.0039(1)

*^a^ U*_eq_ is defined as one-third of the trace of the orthogonalized *U_ij_* tensor.

**Table 3:** Selected bond distances for the Ba_1-*x*_Eu*_x_*Zn_2_Sb_2_ (0.02(1) ≤ *x* ≤ 0.15(1)) system.

Atomic Pair	Bond Distance (Å)			
	Ba_0.98(1)_Eu_0.02_Zn_2_Sb_2_	Ba_0.96(1)_Eu_0.04_Zn_2_Sb_2_	Ba_0.92(1)_Eu_0.08_Zn_2_Sb_2_	Ba_0.85(1)_Eu_0.15_Zn_2_Sb_2_
Ba/Eu–Zn1 (× 2)	3.890(1)	3.885(1)	3.876(1)	3.859(1)
Ba/Eu–Zn2	3.554(2)	3.553(1)	3.550(1)	3.548(1)
Ba/Eu–Zn2 (× 2)	3.864(1)	3.866(1)	3.868(1)	3.873(1)
Ba/Eu–Sb1	3.488(1)	3.487(1)	3.480(1)	3.473(1)
Ba/Eu–Sb1 (× 2)	3.450(1)	3.499(1)	3.496(1)	3.489(1)
Ba/Eu–Sb2 (× 2)	3.700(1)	3.699(1)	3.693(1)	3.686(1)
Ba/Eu–Sb2 (× 2)	3.721(1)	3.717(1)	3.709(1)	3.693(1)
Zn1–Sb1 (× 2)	2.658(1)	2.657(1)	2.656(1)	2.653(1)
Zn1–Sb1	2.816(1)	2.819(1)	2.818(1)	2.673(1)
Zn1–Sb2	2.674(1)	2.673(1)	2.672(1)	2.820(1)
Zn2–Sb1	2.671(2)	2.669(1)	2.667(1)	2.666(1)
Zn2–Sb2 (× 2)	2.718(1)	2.728(1)	2.727(1)	2.726(1)
Zn2–Sb2	2.764(1)	2.767(1)	2.766(1)	2.767(1)

## Data Availability

The original supplementary crystallographic data for this study is openly available in the Cambridge Crystallographic Data Center (12 Union Road, Cambridge CB2 1EZ, UK; fax: +44-1223-336033) at www.ccdc.cam.ac.uk/data_request/cif (accessed on 10 January 2025), or by emailing data_request@ccdc.cam.ac.uk. The reference/accession number to be used is CCDC 2405340-2405343. Furthermore, the raw data supporting the conclusions of this article will be made available by the authors on request.

## Supplementary Materials

The following supporting information can be downloaded at: https://www.mdpi.com/article/10.3390/molecules30020310/s1, Table S1: Structural details of the hypothetical model for BaZn_2_Sb_2_ and Ba_0.75_Eu_0.25_Zn_2_Sb_2_; Figure S1: EDS analysis and elemental mapping results for Ba_0.98(1)_Eu_0.02_Zn_2_Sb_2_. An evaluated EDS composition for this crystal is Ba_0.95_Eu_0.01_Zn_2.06_Sb_1.97_; Figure S2: EDS analysis and elemental mapping results for Ba_0.96(1)_Eu_0.04_Zn_2_Sb_2_. An evaluated EDS composition for this crystal is Ba_0.98_Eu_0.06_Zn_1.97_Sb_2.00_; Figure S3: EDS analysis and elemental mapping results for Ba_0.92(1)_Eu_0.08_Zn_2_Sb_2_. An evaluated EDS composition for this crystal is Ba_0.91_Eu_0.10_Zn_2.00_Sb_1.99_; Figure S4: EDS analysis and elemental mapping results for Ba_0.85(1)_Eu_0.15_Zn_2_Sb_2_. An evaluated EDS composition for this crystal is Ba_0.83_Eu_0.21_Zn_1.95_Sb_2.02_; Figure S5: Temperature-dependent (a) power factor *PF* of the four title compounds in Ba_1-*x*_Eu*_x_*Zn_2_Sb_2_ (0.02(1) ≤ *x* ≤ 0.15(1)) system between 303 and 793 K and (b) lattice thermal conductivity *κ*_latt_ of the four title compounds in the Ba_1-*x*_Eu*_x_*Zn_2_Sb_2_ (0.02(1) ≤ *x* ≤ 0.15(1)) system measured between 323 and 773 K. The experimental data of the reference compound BaZn2Sb2 [24] is also plotted for comparison purposes.; Figure S6: TGA results for the four title compounds in the Ba_1-*x*_Eu*_x_*Zn_2_Sb_2_ (0.02(1) ≤ *x* ≤ 0.15(1)) system over the temperature range between 300 and 1173 K.
