# Supplementary material for: Eu-Substituents-Induced Modifications in the Thermoelectric Properties of the Zintl Phase Ba1-xEuxZn2Sb2 System
Source: Molecules. 2025 Jan 14;30(2):310. doi: 10.3390/molecules30020310 (PMC11767480; doi:10.3390/molecules30020310)
Supplement: Supplementary file 1 [file molecules-30-00310-s001.zip › molecules-3387322-supplementary.pdf]

Eu-Substituents-Induced Modifications in the Thermoelectric Properties of the Zintl Phase $Ba_{1-x}Eu_xZn_2Sb_2$ System

Daewon Shim [1], Junsu Lee [1], Aziz Ahmed [1], Ji Hee Pi [2], Myung-Ho Choi [3], Kang Min Ok [3], Kyu Hyoung Lee [2] and Tae-Soo You [1,*]

[1] Department of Chemistry, Chungbuk National University, Cheongju 28644, Chungbuk, Republic of Korea
[2] Department of Materials Science and Engineering, Yonsei University, Seoul 03722, Republic of Korea
[3] Department of Chemistry, Sogang University, Seoul 04107, Republic of Korea
* Correspondence: tsyou@chungbuk.ac.kr; Tel.: +82-43-261-2282; Fax: +82 (43) 267-2289

Table S1. Structural details for the hypothetical models of $BaZn_2Sb_2$ and $Ba_{0.75}Eu_{0.25}Zn_2Sb_2$

Chemical formula	$BaZn_2Sb_2$	$Ba_{0.75}Eu_{0.25}Zn_2Sb_2$
Structure type	$BaCu_2S_2$-type	
Space group	*Pm* (No.6)	
Unit cell dimensions (Å)	*a* = 10.5498	*a* = 10.524
	b = 4.5053	*b* = 4.482
	c = 11.653	*c* = 11.626
Volume ($Å^3$)	553.82	548.38

Atomic coordinates				
Atom	Wyckoff site	*x*	*y*	*z*
$BaZn_2Sb_2$				
Ba*a*	1*a*	0.2545	0	0.8211
Ba*b*	1*b*	0.7455	0.5	0.1789
Ba*c*	1*a*	0.7545	0	0.6789
Ba*d*	1*b*	0.2455	0.5	0.3211
Sb1*a*	1*a*	0.1529	0	0.5357
Sb1*b*	1*b*	0.8471	0.5	0.4643
Sb1*c*	1*a*	0.6529	0	0.9643
Sb1*d*	1*b*	0.3471	0.5	0.0357
Sb2*a*	1*a*	0.0237	0	0.164
Sb2*b*	1*b*	0.9763	0.5	0.836
Sb2*c*	1*a*	0.5237	0	0.336
Sb2*d*	1*b*	0.4763	0.5	0.664
Zn1*a*	1*a*	0.4461	0	0.1175
Zn1*b*	1*b*	0.5539	0.5	0.8825
Zn1*c*	1*a*	0.9461	0	0.3825
Zn1*d*	1*b*	0.0539	0.5	0.6175
Zn2*a*	1*a*	0.4057	0	0.5481
Zn2*b*	1*b*	0.5943	0.5	0.4519
Zn2*c*	1*a*	0.9057	0	0.9519
Zn2*d*	1*b*	0.0943	0.5	0.0481
$Ba_{0.75}Eu_{0.25}Zn_2Sb_2$				
Ba*a*	1*a*	0.2533	0	0.8185

Eu*b*	1*b*	0.7467	0.5	0.1815
Ba*b*	1*a*	0.7533	0	0.6815
Ba*c*	1*b*	0.2467	0.5	0.3185
Sb1*a*	1*a*	0.1555	0	0.5332
Sb1*b*	1*b*	0.8445	0.5	0.4668
Sb1*c*	1*a*	0.6555	0	0.9668
Sb1*d*	1*b*	0.3445	0.5	0.0332
Sb2*a*	1*a*	0.0243	0	0.1661
Sb2*b*	1*b*	0.9757	0.5	0.8339
Sb2*c*	1*a*	0.5243	0	0.3339
Sb2*d*	1*b*	0.4757	0.5	0.6661
Zn1*a*	1*a*	0.4440	0	0.1157
Zn1*b*	1*b*	0.5560	0.5	0.8843
Zn1*c*	1*a*	0.9440	0	0.3843
Zn1*d*	1*b*	0.0560	0.5	0.6157
Zn2*a*	1*a*	0.4083	0	0.5474
Zn2*b*	1*b*	0.5917	0.5	0.4526
Zn2*c*	1*a*	0.9083	0	0.9526
Zn2*d*	1*b*	0.0917	0.5	0.0474

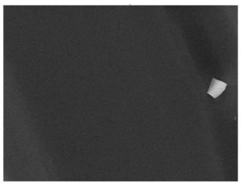

Element	Wt%	Wt% Sigma	Atomic %
Ba	25.64	0.11	18.95
Eu	0.42	0.08	0.28
Zn	26.58	0.11	41.28
Sb	47.36	0.11	39.49

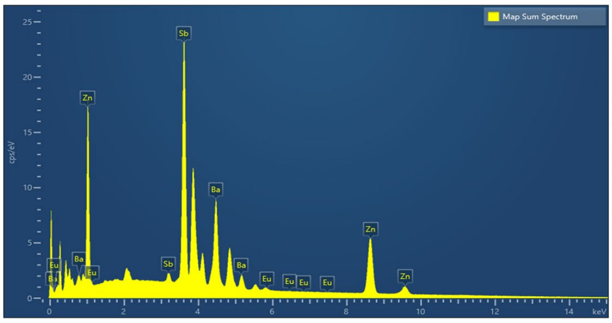

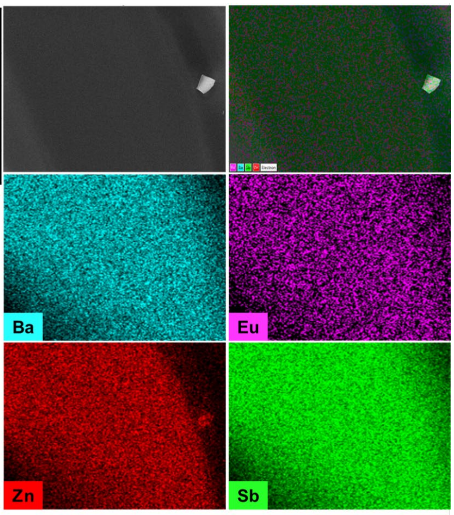

Figure S1. EDS analysis and elemental mapping results for $Ba_{0.98(1)}Eu_{0.02}Zn_2Sb_2$. An evaluated EDS composition for this crystal is $Ba_{0.95}Eu_{0.01}Zn_{2.06}Sb_{1.97}$.

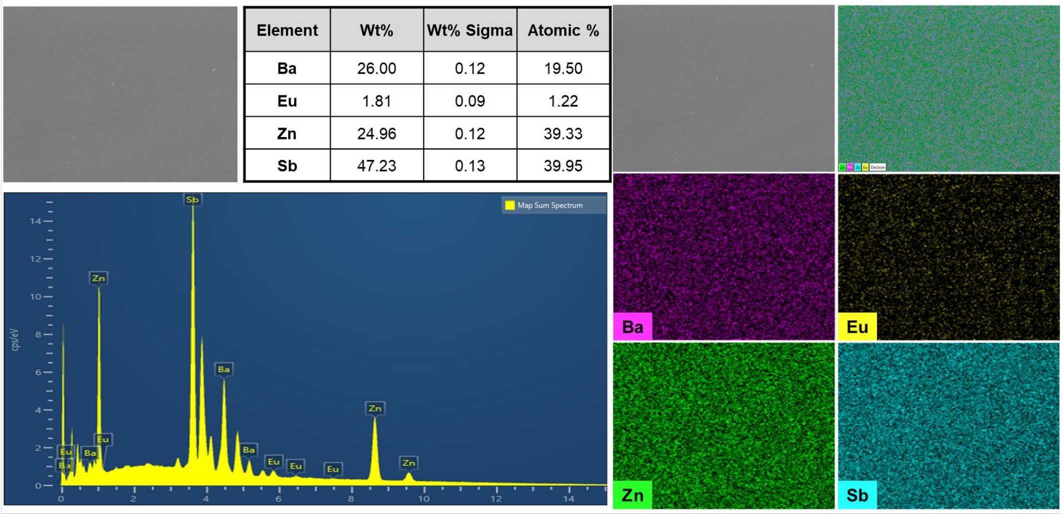

Element	Wt%	Wt% Sigma	Atomic %
Ba	26.00	0.12	19.50
Eu	1.81	0.09	1.22
Zn	24.96	0.12	39.33
Sb	47.23	0.13	39.95

Figure S2. EDS analysis and elemental mapping results for $Ba_{0.96(1)}Eu_{0.04}Zn_2Sb_2$. An evaluated EDS composition for this crystal is $Ba_{0.98}Eu_{0.06}Zn_{1.97}Sb_{2.00}$.

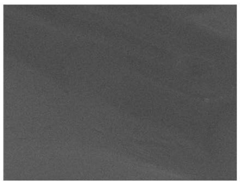

Element	Wt%	Wt% Sigma	Atomic %
Ba	24.46	0.10	18.29
Eu	2.91	0.08	1.97
Zn	25.41	0.10	39.91
Sb	47.22	0.11	39.83

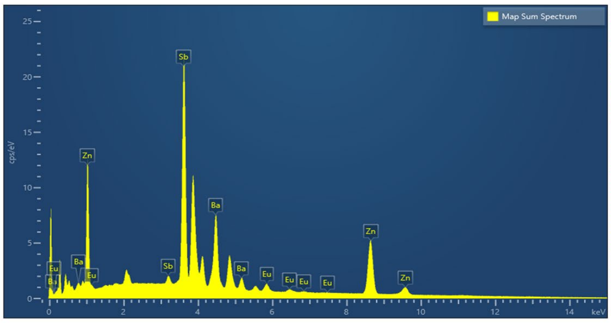

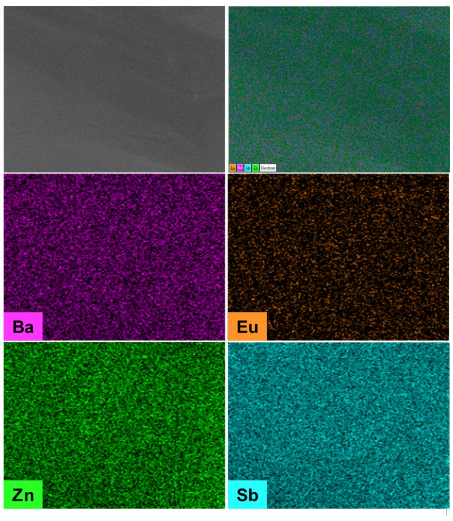

Figure S3. EDS analysis and elemental mapping results for $Ba_{0.92(1)}Eu_{0.08}Zn_2Sb_2$. An evaluated EDS composition for this crystal is $Ba_{0.91}Eu_{0.10}Zn_{2.00}Sb_{1.99}$.

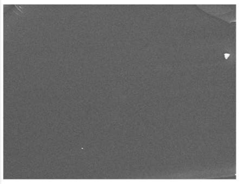

Element	Wt%	Wt% Sigma	Atomic %
Ba	21.88	0.08	16.51
Eu	6.07	0.07	4.14
Zn	24.54	0.09	38.91
Sb	47.51	0.09	40.44

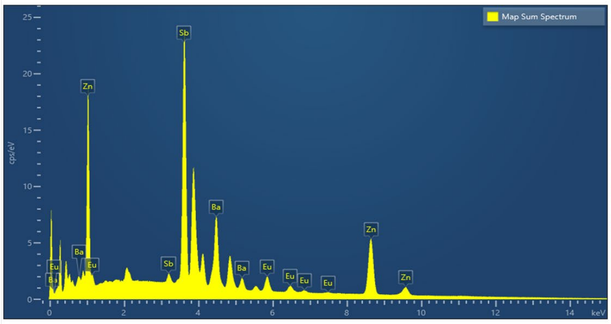

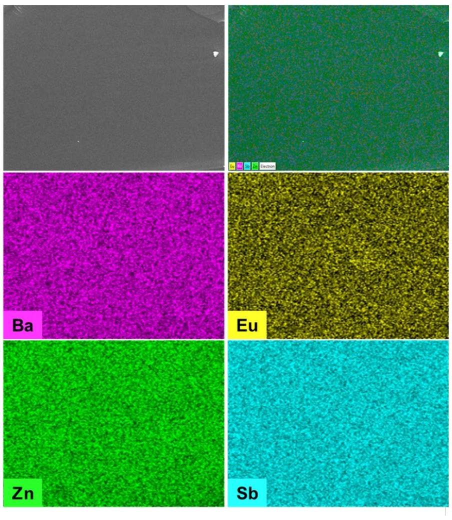

Figure S4. EDS analysis and elemental mapping results for $Ba_{0.85(1)}Eu_{0.15}Zn_2Sb_2$. An evaluated EDS composition for this crystal is $Ba_{0.83}Eu_{0.21}Zn_{1.95}Sb_{2.02}$.

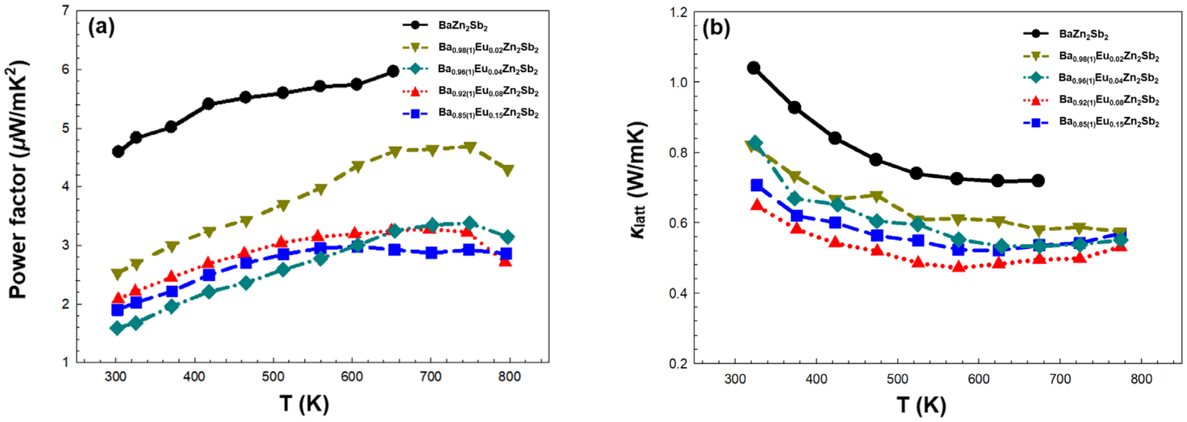

Figure S5. Temperature-dependent (a) power factor *PF* measured between 303 and 793K and (b) lattice thermal conductivity κ_{latt} measured between 323 and 773K for the four title compounds in the $Ba_{1-x}Eu_xZn_2Sb_2$ ($0.02(1) \leq x \leq 0.15(1)$) system. The experimental data for the reference compound $BaZn_2Sb_2$ [24] is also plotted for comparison purposes.

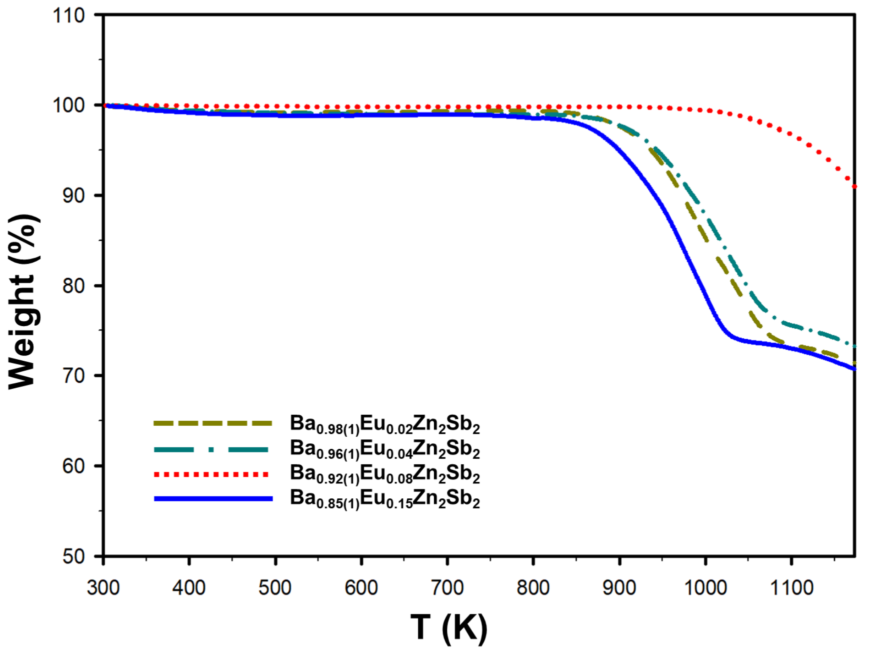

Figure S6. TGA results for the four title compounds in the $Ba_{1-x}Eu_xZn_2Sb_2$ ($0.02(1) \le x \le 0.15(1)$) system over the temperature range between 300 and 1173 K.
